# Impact of Reductive Stress on Human Infertility: Underlying Mechanisms and Perspectives

**DOI:** 10.3390/ijms252111802

**Published:** 2024-11-02

**Authors:** Efthalia Moustakli, Athanasios Zikopoulos, Charikleia Skentou, Periklis Katopodis, Ekaterini Domali, Anastasios Potiris, Sofoklis Stavros, Athanasios Zachariou

**Affiliations:** 1 Laboratory of Medical Genetics, Faculty of Medicine, School of Health Sciences, University of Ioannina, 45110 Ioannina, Greece; 2 Obstetrics and Gynecology, Royal Devon and Exeter Hospital Barrack Rd, Exeter EX 25 DW, UK; thanzik92@gmail.com; 3 Department of Obstetrics and Gynecology, Medical School of Ioannina, University General Hospital, 45110 Ioannina, Greece; haraskentou@gmail.com; 4 First Department of Obstetrics and Gynecology, Alexandra Hospital, Medical School, National and Kapodistrian University of Athens, 11528 Athens, Greece; kdomali@yahoo.fr; 5 Third Department of Obstetrics and Gynecology, University General Hospital “ATTIKON”, Medical School, National and Kapodistrian University of Athens, 12462 Athens, Greece; apotiris@med.uoa.gr (A.P.); sfstavrou@med.uoa.gr (S.S.); 6 Department of Urology, School of Medicine, University of Ioannina, 45110 Ioannina, Greece; zahariou@otenet.gr

**Keywords:** antioxidants, reductive stress, fertility, sperm, oocyte, side effects, hormesis

## Abstract

Antioxidants have a well-established effect on general health and are essential in preventing oxidative damage to cells by scavenging free radicals. Free radicals are thought to be neutralized by these substances, which include polyphenols, β-carotene, and vitamins C and E, reducing cellular damage. On the other hand, recent data indicates that consuming excessive amounts of antioxidants may have side effects. Apoptosis and cell signaling are two beneficial physiological processes that are affected by excessive supplementation. Other negative effects include paradoxical enhancement of oxidative stress and unbalanced cellular redox potential. Overdosing on particular antioxidants has been associated with increased medication interactions, cancer progression, and fatality risks. Additionally, the complex impacts they may have on fertility might be both useful and adverse, depending on the quantity and duration of usage. This review delves into the dual role of antioxidants and emphasizes the importance of employing antioxidants in moderation. Antioxidant overconsumption may disrupt the oxidative balance necessary for normal sperm and oocyte function, which is one of the potential negative effects of antioxidants on fertility in both males and females that are also investigated. Although modest usage of antioxidants is generally safe and useful, high levels of antioxidants can upset hormonal balance, impair sperm motility, and negatively impact the outcomes of assisted reproductive technologies (ART). The findings emphasize the need to use antioxidant supplements in a balanced way, the importance of further research to optimize their use in fertility treatments, and the importance of supporting reproductive health to avoid adverse effects.

## 1. Introduction

Reactive oxygen species (ROS) are oxygen-based compounds that are extremely unstable and reactive due to the fact that they have free, unpaired electrons in their outer orbit. These include the hydroxyl radical, hydrogen peroxide, and superoxide anion [1]. ROS are naturally produced in the mitochondria as a by-product of aerobic metabolism; they are 1% to 5% of the oxygen that has been metabolized. These byproducts of cell metabolism are injurious and are normally liberated from the electron transfer chain and other cellular systems [2]. Biological components that might suffer from damage include proteins, lipids, and nucleic acids due to ROS. Their high reactivity can lead to DNA damage, protein peroxidation, and lipid peroxidation, respectively. ROS half-lives commonly fall in the range of nanoseconds [3].

Aging, neurological diseases, cancer, and infertility are only a few of the disorders that ROS has been linked to. It is estimated that OS, in which high levels of ROS interfere with male reproductive functions, is one of the major causative agents [4]. Infertility affects approximately 17.5% of the total global adult population, with 10–15% of couples experiencing infertility. In 40–50% of these cases, the causes are solely or partly due to male factors, while 10–15% of couples experience delayed conception [5,6]. At higher concentrations, ROS significantly impair cellular mechanisms, but they also play a vital physiological role at low concentrations in sperm maturation, embryogenesis, immune response against infections, cellular signaling, and cellular growth [5]. Human spermatozoa require capacitation-related tyrosine phosphorylation to fertilize oocytes, while superoxide dismutase (SOD) scavenges superoxide radicals, preventing oxidative damage that could impair sperm function [6].

Antioxidants are chemical substances acting as modulators of antioxidant defense and, therefore, capable of removing ROS by-products. Additionally, they can combine covalently with any other molecule except reactive nitrogen species (RNS), which leads to aberrant development and subsequent cell lesions. [7]. The body has both enzymatic and non-enzymatic forms of natural antioxidants, considered endogenous antioxidants. Exogenous antioxidants, on the other hand, include vitamins and trace elements, which are essential to maintain the proper balance between the processes of oxidation and reduction in the cell. Exogenous antioxidants are widely available, reasonably priced, and non-prescription treatment modalities [8].

**Figure 1:**
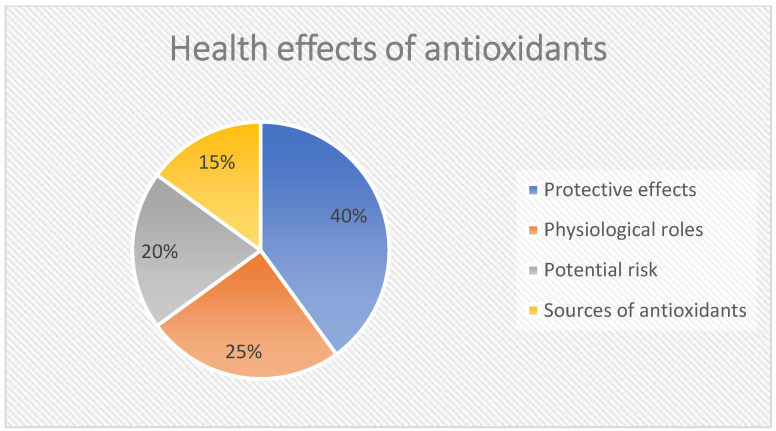
This pie chart illustrates the multifaceted health effects of antioxidants, highlighting their protective roles in preventing aging, inflammation, and cancer. Understanding these dynamics is crucial for informed health decisions regarding antioxidant consumption. In detail: Protective effects: Prevents aging, reduces inflammation, lowers cancer risk. Physiological roles: Supports sperm maturation, aids in embryogenesis, enhances immune response. Potential risk: Disrupts redox equilibrium and may lead to reductive stress. Sources of antioxidants: Endogenous (produced by the body) and exogenous (vitamins from diet).

Antioxidants can prevent aging, inflammation, and cancer by preventing the generation of reactive species and its adverse effects. They act as repair enzymes, radical scavengers, preventative agents, and adaptation agents, amongst others. Redox equilibrium is necessary to maintain a healthy cellular microenvironment [9]. Studies have indicated that disrupting this fragile balance can have adverse consequences, and antioxidant supplements have not always proven beneficial [10,11,12]. However, the increasing practice of antioxidant intake, in particular as dietary supplements, resulted in a complicated and somewhat paradoxical process known as the ‘antioxidant paradox’ [13]. Inversely, high antioxidant levels may enhance imbalances in redox homeostasis, disturbing cellular processes. They additionally may generate a reductive ambiance that may further lead to reductive stress and its consequences on hormesis and cellular stress responses. This inconsistency highlights the need for a more accurate conception of the impact of antioxidants on human health and challenges the oversimplified belief of their unquestionable benefit (Figure 1).

This study aims to investigate the consequences of excessive antioxidant consumption on human health and to explore the antioxidant paradox. Additionally, it seeks to elucidate how excessive antioxidants can shift from being protective agents to potential hazards by examining the molecular and cellular mechanisms underlying these effects. Our goals are to improve public health recommendations and enable people to make more informed decisions about their antioxidant consumption through this research.

## 2. Reductive Stress Generation and Notable Adverse Effects

Oxidative stress (OS) is closely linked to aging and arises from an imbalance between oxidants and antioxidants. An overabundance of antioxidants can lead to a condition known as reductive stress (RS), characterized by an excess of reducing agents that disrupts the fine redox balance [14]. Many medical fields now acknowledge the role of RS as a contributor to numerous pathological disorders [15].

Redox regulators and mediators are interconnected and influence various biological processes. For instance, overexpression of antioxidant enzymes decreases the levels of ROS, which may potentially lead to increased levels of hydrogen peroxide (H_2_O_2_) and interfere with redox homeostasis [16]. High cell levels of nicotinamide adenine dinucleotide hydrogen (NADH) can lead to a collapse in cellular homeostasis, increase mitochondrial oxidation, and disturb protein folding [17].

Interestingly, appropriate disulfide bond formation and protein folding depend on mitochondrial ROS and the RS-mediated reduction of these ROS [2]. However, OS induced by persistent RS may create a vicious cycle that exacerbates the condition. Excessive reducing equivalents can impair cellular growth responses, render mitochondrial function vulnerable, and disrupt metabolic pathways [18]. Cells possess a complex antioxidant defense system to regulate ROS levels, maintaining homeostasis through the regulation of reactive oxygen and nitrogen species (RONS) levels. Imbalances in RONS signaling may lead to oxidative damage, potentially resulting in various health issues.

Exogenous RONS scavengers interfere with normal signaling and even attenuate some of the beneficial effects of RNS [7]. For instance, vitamin C has been shown to inhibit key pathways such as the inositol triphosphate system and the Nrf2 activation pathway, as well as blunt transient elevations in RONS. Restoring optimal RONS concentrations may offer novel approaches to therapy for diseases associated with ROS [19].

However, excessive application of antioxidants disrupts the redox balance and may lead to adverse effects in humans and animals [20]. Overuse has been associated with diseases such as cardiomyopathy, cancer, Alzheimer’s disease, and anomalies in embryogenesis [21]. Proper regulation of the cellular redox system is essential, especially during embryogenesis, since high levels of antioxidants have been associated with teratogenicity, manifesting as malformations and slowed development [22].

In the context of fertility, however, asynchronous chromosomal condensation is associated with increased sperm DNA decondensation, which can reduce fertility. Such negative effects could be attributed to vitamin C’s ability to break the disulfide bonds in sperm protamines. Additionally, vitamin C influences both sperm motility and lipid peroxidation in a dose-dependent manner [23]. Similar results can be observed with other antioxidant supplements. Further research is required to determine the interrelationship between redox balance and fertilization. For this reason, clinicians should conduct comprehensive assessments of their redox statuses before administering antioxidants to patients to prevent overdosing [24].

**Figure 2:**
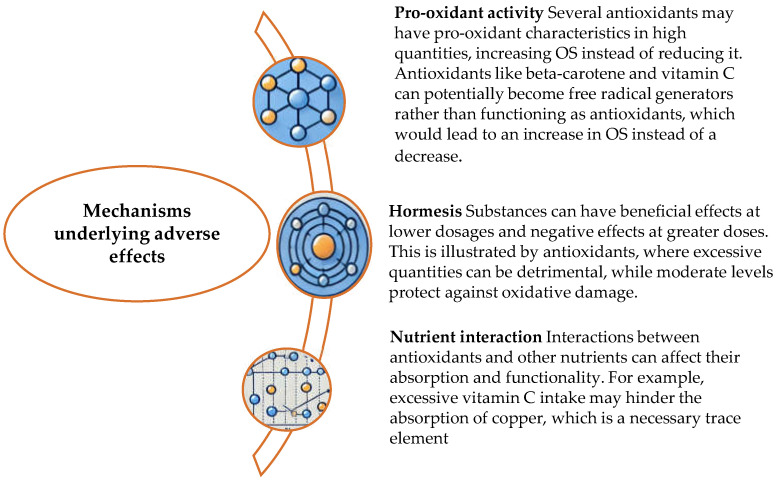
Mechanisms that conceal detrimental consequences and how they function are shown in the table.

The therapeutic potential of various antioxidants, including vitamins, enzymes, nitroxide radical compounds, SOD mimics, and non-enzymatic antioxidants, has been explored [25]. However, clinical research indicates that these antioxidants often provide minimal benefits, pose serious adverse effects at high doses, and fail to halt the progression of ROS-related diseases [26]. Factors such as high renal clearance, poor bioavailability, metabolite toxicity, and inadequate target selectivity may contribute to these suboptimal outcomes [27] (Figure 2).

## 3. Reductive Stress Hypothesis in Cells and Its Association with Male Infertility

The respiratory chain and tricarboxylic acid cycle are two of the vital metabolic pathways that generate ROS, including O_2,_ which is an important regulator of cellular activity but can also cause impairment in cellular activity and produce OH•. Enzymatic antioxidants like catalase, glutathione peroxidase (GPx), and peroxidase (Prx) can convert hydrogen peroxide (H_2_O_2_) into water (H_2_O). However, GPx needs reduced glutathione (GSH) to act intracellularly [27]. The methionine cycle and trans-sulfuration pathway synthesize cysteine, an amino acid that contains sulfur and is necessary to form GSH [25]. Overconsumption of these compounds-especially with vitamin supplementation- may result in the overproduction of GSH and hence cause an imbalance in intracellular redox status [26].

Male infertility may result from increased levels of GSH in damaged mitochondria, which impairs NADH oxidation and leads to reductive stress. This imbalance hinders the maintenance of cellular reducing capacity, ultimately generating excess NADPH. Furthermore, impaired function of mitochondrial complex I reduces the OS level in gametes and increases NADH concentration [4]. This kind of imbalance between the process of oxidation and reduction could be linked to varicocele, idiopathic male infertility, and poor sperm motility [28].

Antioxidants have been used in the treatment of diseases associated with OS, yet overdosing or taking the wrong kind of antioxidants orally still occurs frequently [26]. Only patients with higher seminal ROS levels had improved sperm function, according to the study. For instance, antioxidant treatment can be of benefit in decreasing ROS overproduction and related OS in male infertility due to varicocele [29]. Nonetheless, one critical review and meta-analysis that similarly revealed no appreciable variations in the pregnancy rate discovered conflicting results for sperm morphology, concentration, motility, and DNA fragmentation [30].

Protamines replace nucleosome-bound DNA during the chromatin remodeling process that turns spermatids into spermatozoa [31]. Enzyme-induced transient double-strand breaks have the potential to initiate a damage response. Additional elements critical to the removal of TOP2B complexes from DNA and the repair of DNA breaks include tyrosyl-DNA phosphodiesterase 1 (TDP1) and poly (ADP-ribose) polymerase (PARP). Changes in PARP activity under reductive stress caused by elevated GSH/GSSG and NAD(P)H/NAD(P)+ ratios significantly affect DSB repair activity [32] (Table 1).

## 4. Effects of Reductive Stress and Oxidative Damage on Sperm

Recent advancements in sperm function testing and DNA integrity assessment have underlined the value of sperm DNA as a potent predictor of fertilization success, pregnancy, and embryo development. Sperm DNA is particularly susceptible to damage from OS, primarily due to inadequate protamination, leading to fragmentation characterized by single- and double-strand breaks in the sperm genome [33]. Hydroxyl radicals attach to both nucleus and mitochondrial DNA bases and create an extensive array of oxidation products that negatively affect DNA template integrity. Unrepaired 8-OHdG in paternal DNA may be passed onto future generations and impact the embryo development [34].

Infertile individuals’ sperm DNA fragmentation is caused mainly by OS. Antioxidants represent an increasingly common front-line treatment that is usually undertaken prior to assisted conception [4]. According to a study, daily antioxidant treatment with vitamins C and E, β-carotene, zinc, and selenium reduced sperm DNA damage [35]. Nevertheless, inherent variables that cause DNA fragmentation include defective chromatin packing and premature apoptosis [22]. In individuals with extremely high levels of DNA fragmentation, high intakes of antioxidants may be detrimental. Overdosing cells with antioxidants can interfere with cellular processes such as protein folding and the formation of bisulfide bonds [24]. High levels of antioxidants stimulate the activity of redox-sensitive transcription factors and alter gene expression patterns, resulting in retardation of development and deformities [36]. Human spermatozoa may eventually develop DNA damage as a result of the elevated NADH/NAD+ ratio in RS, which also leads to excessive ROS generation in mitochondria and overflows H_2_O_2_ into the cytoplasm [34].

## 5. Antioxidants in Oxidative Stress-Induced Male Infertility

Antioxidant therapy is gaining popularity as the population ages rapidly. Reducing OS is believed to help shield against certain diseases [35]. Studies have identified that antioxidant therapy has the ability to enhance male reproductive health [10,23]. However, excessive supplementation has revealed their potential side effects. Antioxidants serve as a line of defense against OS by acting as reducing agents to buffer ROS [37].

Semen provides an optimal environment for sperm with regards to pH, viscosity, nutritional content, and antioxidants that facilitate fertility due to the seminal plasma derived from the prostate, bulbourethral glands, and seminal vesicles. Sperm cultured in vitro without seminal plasma show a substantial increase in OS indicators and a corresponding decrease in motility [38].

Oxygen metabolism produces ROS, which are unstable and highly reactive molecules that can disrupt cellular function and lead to abnormal reproductive outcomes [24,39]. Nevertheless, ROS also exerts an important role in critical reproductive events such as sperm maturation, hyperactivation, capacitation, acrosome reaction, and embryonic morphogenesis. De Lamirande et al. [40] highlighted the role of superoxide anion (O^2−^) in initiating early sperm capacitation through two key observations. First, as soon as capacitation was initiated, spermatozoa began generating O^2−^; over the course of the next several hours, these levels gradually decreased. Second, capacitation persisted even after the addition of SOD, an O^2−^ scavenger. The findings suggest that spermatozoa require elevated levels of O^2−^ to trigger hyperactivation, which can only be sustained by a subsequent basal level of this free radical. Indeed, SOD has been utilized to restore hyperactivation to spermatozoa.

Additionally, scavenging of superoxide by SOD also inhibits tyrosine phosphorylation associated with capacitation in human sperm [40]. Cells utilize both endogenous and exogenous antioxidant processes to scavenge excess ROS. In this context, non-enzymatic antioxidants such as vitamins A, C, and E, L-carnitine, coenzyme Q10, and trace minerals play essential roles [24]. The most active neutralizing vitamins of toxic by-products in the body are vitamins C and E. Dietary sources of vitamin C do not appear to pose a similar risk, but high intake of vitamin E has been related to increased high-sensitivity C- reactive protein (hs-CRP) levels along with other negative outcomes such as increased OS, lipid peroxidation, and blunted exercise-induced adaptations [41].

Endogenous antioxidants include enzymes such as catalase and thiol peroxidases. Key enzymatic antioxidants involved in infertility include GPX, CAT, and SOD [42]. GPX utilizes glutathione as a coenzyme that protects sperm against DNA damage and lipid peroxidation. Superoxide anions are scavenged by SOD, which is changed to H2O2 and O_2_. Sertoli cells have high SOD levels, and during spermatogenesis, germ cells are very active [43]. Vitamin C also plays a role in the prevention of DNA damage in sperm, and individuals suffering from asthenozoospermia show lower seminal levels of CAT. Other antioxidants, such as zinc, tocopherol, vitamin E, and selenium, provide defense against lipid oxidation. Although lycopene has been found absent in semen, it is still capable of making tissues resistant to ischemia-reperfusion damage. Increased sperm motility and count have been linked to lower ROS levels in the presence of ubiquinol, which is found in seminal plasma [12].

## 6. Influence of Redox Balance on Ovarian Function

As a prerequisite for the ovaries to function normally, the redox equilibrium must be maintained, and antioxidants play a significant role in this process. However, the precise functions and chemical processes of antioxidants and their complex interplay with ROS are still unclear. This underscores the need for future research to determine whether antioxidant supplements can prevent reproduction-related disorders [44]. ROS influences a wide range of physiological and pathological processes of the ovaries, including oocyte maturation, ovarian steroid production, and follicular development. ROS may exert a variety of roles within the follicular fluid in these processes [44,45].

During the preovulatory phase, prostaglandins, cytokines, proteolytic enzymes, and hormones surge in the follicles, leading to ROS generation that cumulatively results in follicle rupture and alterations in blood flow. ROS also acts during the luteal phase to synthesize progesterone. One of the main processes that produce ROS is steroidogenesis, and during pregnancy, a decrease in SOD1 causes an increase in ROS generation [46]. Membrane disruption, DNA damage, protein oxidation, and lipid peroxidation are four key mechanisms through which OS damages cells. Substance abuse, alcohol consumption, tobacco usage, and psychological stress all result in increased ROS production [7]. ROS regulates cyclic endometrial alterations, hormone signaling, and tissue remodeling during the menstrual cycle. In the ovaries, ROS and antioxidants in the follicular fluid may impact important reproductive events such as egg quality, fertilization, implantation, and embryo development [47]. Through changes in ovulation, steroidogenesis, oocyte maturation, and the acceleration of granulosa cell death, OS can impair the function of the female genital tract. Indeed, higher ROS levels have been reported to be higher in infertile women, which may serve as evidence for the linkage between OS and infertility [48].

ROS and OS are believed to take part in the pathophysiology of female reproductive disorders endometriosis, hydrosalpinx, polycystic ovarian syndrome (PCOS), and unexplained subfertility [49]. Proposed mechanisms of action for supplemental antioxidants include enhanced endometrial blood circulation, reduced hyperandrogenism, decreased insulin resistance, fertile cervical mucus, and effects on prostaglandin production and steroidogenesis [47].

## 7. Antioxidants and Reproduction

One popular therapeutic approach for male infertility is antioxidant supplementation; however, it is critical to differentiate between dietary and over-the-counter antioxidant supplements [50]. Antioxidants function by donating electrons to neutralize oxidants, which are molecules that remove electrons and cause oxidative damage. Maintaining homeostasis requires a balance between ROS, free radical formation, and antioxidant levels in the body’s redox potential. However, antioxidant supplements have not always shown consistently positive results, and excessive intake can be harmful. Studies have linked antioxidants to improved sperm quality and pregnancy outcomes, though the effects on live birth rates remain inconclusive [51]. Some experts argue that these interventions may have little to no effect on sperm development or normal physiological functions and may even worsen semen quality [52].

In human embryos, elevated antioxidant levels have been associated with beneficial outcomes such as enhanced cellular compaction and blastulation due to glycolytic energy production [53]. The metabolic changes that occur may alter the expression of redox-sensitive transcription factors and genes. Proper ROS production and antioxidant scavenging are vital for maintaining the delicate cellular redox balance, which is crucial for physiological activities such as male reproduction regulation [54].

Antioxidants neutralize free radicals, reducing their harmful effects. However, in the female genital tract, excessive ROS can impact processes like ovulation, apoptosis, steroidogenesis, and oocyte maturation. For instance, a deficiency of ovarian glutathione can accelerate atresia in antral follicles [44]. Psychological stress, which leads to a buildup of ROS, can decrease oocyte quality and disrupt granulosa cell function. Interestingly, injecting antioxidants into female mice’s ovaries resulted in a sharp decline in ovulation [55], suggesting that ROS, triggered by luteinizing hormone, may act as intermediaries in the ovulation process. Further research is needed to explore the mechanisms behind these effects and how antioxidant intake via food or drink might influence ovulation in humans [55].

Some antioxidants could have contraceptive effects or help women struggling with infertility, according to emerging studies. Further research is required to clarify the link between antioxidant supplementation and female fertility outcomes [56]. Synthetic phenolic antioxidants, such as butylated hydroxyanisole (BHA), butylated hydroxytoluene (BHT), and the gallic acid esters propyl, octyl, and dodecyl gallate, are commonly used as vitamin E substitutes. These synthetic antioxidants are primarily used to extend product shelf life [57]. However, they have been associated with liver toxicity, carcinogenic effects, and hormonal disruptions. Ethylhexylglycerin, a known skin irritant, is used as a skin conditioner, while phenoxyethanol serves as a preservative but is also a skin allergen [58]. Although research on the specific effects of BHT on fertility is still ongoing, some studies suggest that exposure to synthetic antioxidants like BHT may harm reproductive health. The most frequently studied beta-hydroxy acid (BHA) in relation to pregnancy is salicylic acid, a component of aspirin. High oral doses of salicylic acid have been shown to cause birth defects and other pregnancy-related complications [59].

## 8. Cells-Defending Enzymes Against Radical Damage

Although it has limited regulation in follicular growth and differentiation, catalase is essential for the metabolism of ROS. It is minimally expressed in oocytes and is primarily located in peroxisomes. Inhibiting catalase can cause chromosomal abnormalities, but it can also protect the genome from oxidative damage during meiosis. Studies have shown that catalase activity is higher in larger follicles in mammals such as pigs, goats, and rats [60]. Enhanced activity throughout ovarian development and luteinization is reported in rat ovarian granulosa and theca cells. Although the amount of catalase in follicular fluid varies among follicles, its concentration remains consistent throughout various estrous phases [61].

Gonadotropin regulation may be linked to the distribution and oscillation of catalase during ovarian cycles. Follicle-stimulating hormone (FSH) and other gonadotropins are essential for follicular maturation, differentiation, and steroidogenesis [62]. Following FSH stimulation, catalase activity increases, particularly in larger follicles. This suggests a potential role in preventing apoptosis and aiding in follicle selection. Moreover, once ovulation is inhibited, catalase activity rises in both the follicle and theca cells [63]. Catalase activity showed a positive correlation with the levels of two components of the steroidogenic electron transport chain, cytochrome P450scc, and ferredoxin, in the ovaries of rats and pigs. Thus, catalase acts as a safeguard in the ovaries, maintaining the balance between ROS and appropriate steroid levels [64].

Mammalian ovaries and other tissues contain several families of SOD enzymes. Immunohistochemistry was used to identify their location within the human body [61]. Theca cells and secondary follicles contain SOD1 and SOD2, respectively, while primordial and primary follicles do not contain these enzymes. Theca and luteinized granulosa cells have been shown to express both SOD isoforms at high levels [65].

Interestingly, large antral follicles exhibit lower SOD activity compared to small and medium-sized follicles, with activity peaking during the proestrus phase. Low human fertilization rates were found to be linked with high SOD activity in follicular fluid. For proper cellular function, SOD must be maintained in the follicles at a specific concentration and activity level to ensure an equilibrium between O^2•−^ and H_2_O_2_. SODs are particularly active in the corpus luteum after ovulation [66]. SOD1 activity is directly associated with progesterone release, while SOD2 primarily protects luteal cells from inflammation-induced oxidative damage. During regression, ROS levels rise in conjunction with a decrease in SOD1 activity [67]. A specific concentration of SOD is necessary to protect oocytes from OS and to maintain the appropriate ROS levels required for ovulation [68].

SOD isoforms in oocytes can be analyzed in vitro using cumulus cell-oocyte complexes (COCs). The DNA or transcription of redox-sensitive genes may be regulated by SOD1 and SOD3, which are expressed in the nucleus and zona pellucida, respectively [66]. SOD2, a scavenger of mitochondrial superoxide, is present in mammalian oocytes. For future processes like ovulation, fertilization, and early embryonic development, COCs might accumulate SODs [69]. OS is significantly heightened during ovulation, leading to increased ROS production. Matzuk et al. found that the fertility of SOD1 knockout mice was reduced. Another team developed a copper chaperone for mice lacking the enzyme SOD). Mice deficient in SOD2 died three weeks after birth. Following the transplantation of the ovaries from the postnatal SOD6-deficient mice, the phases of folliculogenesis were identified [70].

In IVF patients, a positive correlation was found between intrafollicular estradiol levels and SOD enzyme activity [71]. SOD has inhibitory effects on estrogen production. In bovine corpora lutea, luteinizing hormone (LH) can increase the levels of SOD1, SOD2, and catalase. LH-induced overexpression of antioxidant enzymes enhanced corpus luteum function and improved cell viability in sheep [72] (Table 2).

## 9. Disturbance of Redox State Under Pathological Conditions and Ageing

OS may inflict damage on the reproductive systems by diminishing the capacity of antioxidants to scavenge, leading to an increase in the generation of ROS. This imbalance may occur in the pathogenesis of infertility, pregnancy complications, and other reproductive disorders. Additionally, reduced efficacy in antioxidant systems is linked to age-related declines in fertility [72].

Polycystic ovarian syndrome (PCOS) is a common reproductive disorder characterized by the presence of polycystic ovaries, decreased fertilization rates due to ovulation dysfunction and menstrual irregularities, and hyperandrogenism. The primary causes of PCOS include insulin resistance, decreased GSH levels, and mitochondrial dysfunction. Low levels of antioxidants may also contribute to insulin resistance, elevated androgens, and a pro-inflammatory environment [73].

Infertility is defined as the inability to conceive after at least one year of unprotected sexual intercourse. Approximately 15% of couples experience infertility without an apparent cause. Unexplained infertility may be linked to elevated ROS and reduced antioxidant levels [74]. Abortion, poor placentation, fragmentation of embryos, and unsuccessful implantation are all possible outcomes of excessive ROS production. The corpus luteum, necessary for the maintenance of pregnancy in the early stages, is particularly vulnerable to OS [46].

**Figure 3:**
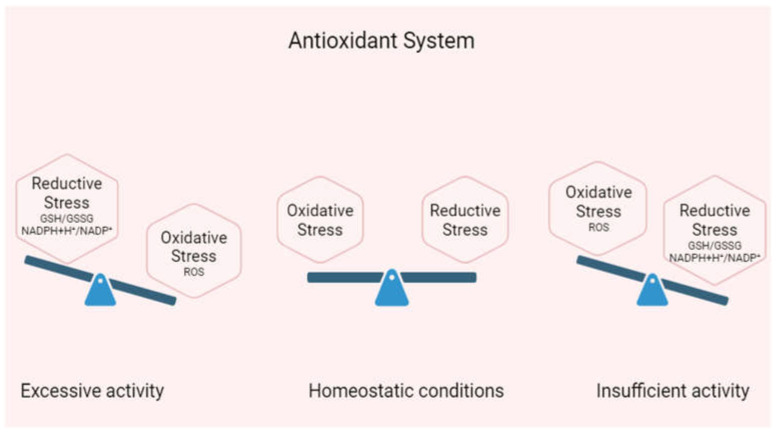
Supplementation with antioxidants in excess is shown in the figure. Redox balance is regulated by Nrf2. Basal Nrf2 activity ensures ideal levels of ROS and reduces antioxidants/agents, enabling redox signaling in homeostatic settings. Reductive stress is brought on by excess Nrf2 activity, which raises the concentration of reducing agents and antioxidants, whereas insufficient Nrf2 activity raises ROS levels, which results in OS.

As a woman ages, the antioxidant levels in their follicular fluid decrease, resulting in diminished fertilization and blastocyst development rates. In aging women, there have been reductions in SOD and catalase levels; mice showed similar losses in both the cytosolic and mitochondrial antioxidants [75] (Figure 3). The oxidative damage associated with aging may adversely affect ovarian function. Notably, fertilization outcomes are associated with the antioxidant capability of neutralizing ROS [68].

## 10. Discussion

The potential health benefits of antioxidants, particularly their ability to protect cells from OS brought on by free radicals, are generally acknowledged. Antioxidants are phytochemicals commonly found in food products such as fruits, vegetables, and other plants, as well as vitamins C and E, beta-carotene, and selenium. While the benefits of antioxidants are frequently emphasized, it is crucial to recognize that they can also have adverse effects that might affect overall health and specific areas, such as fertility [36].

Antioxidants are intended to counteract OS; however, an overabundance of them can disrupt this delicate balance and lead to “reductive stress”. Unbalanced oxidative states pose significant risks to overall health, potentially hindering cellular processes and contributing to diseases such as insulin resistance, heart disease, and certain types of cancer.

It is essential to distinguish between dietary antioxidants and generic supplements. Dietary antioxidants play a vital role in maintaining homeostasis, balancing ROS, free radical production, and antioxidant levels. Deficiencies in vitamins E and C can result in increased OS and DNA damage. In such cases, antioxidant supplements have not consistently proven effective. Recent research suggests that antioxidant supplements could be risky and have unintended negative effects on health. The potential side effects of antioxidant supplements in various clinical scenarios remain unclear. This underscores the importance of relying on dietary antioxidants, found in a balanced diet rich in fruits, vegetables, and other plant-based foods, to maintain optimal health.

The effects of antioxidants on fertility are complex. While moderate antioxidant intake can reduce oxidative damage to sperm DNA and improve sperm quality in men, excessive supplementation may have the opposite effect. On the other hand, excessive supplementation of antioxidants can disrupt the delicate balance of ROS necessary for optimal sperm function, potentially lowering sperm motility and viability. ROS-induced sperm destruction accounts for 30% to 80% of male infertility cases. Moreover, specific antioxidants can adversely affect fertility in both men and women when consumed excessively [50]. Evidence indicates that excessive intake of antioxidants such as vitamins C and E may impair sperm motility and quality in men, leading to decreased fertility. Overconsumption of antioxidants may negatively impact female fertility by disrupting the delicate hormonal balance required for normal menstruation and ovulation.

Furthermore, mammalian embryo development has been linked to elevated antioxidant levels, and glycolytic energy creation seems advantageous for cellular compaction and blastulation. Metabolic changes may alter the expression of redox-sensitive transcription factors and genes [76]. This highlights the current lack of understanding regarding the mechanisms behind the action of antioxidant therapies. These results highlight the importance of giving antioxidant dosage considerable thought when caring for an individual’s reproductive health.

Antioxidant supplementation within the realm of ART, particularly in IVF, is still an active area of research. Some studies suggest that antioxidants may improve outcomes by protecting gametes and embryos from oxidative damage. However, excessive doses may disrupt the delicate balance of oxidation necessary for optimal fertilization and embryo development. It is worth noting that overconsumption of certain antioxidants during pregnancy could potentially lead to teratogenic effects, although this is less common. For example, high doses of vitamin A (as retinoids) have been associated with congenital abnormalities.

While antioxidants are undeniably crucial for preserving health and guarding against OS, their consumption must be managed with utmost care. An overabundance can be detrimental to overall health and fertility, underscoring the importance of responsible and balanced intake.

## 11. Conclusions

Antioxidants have dose-dependent effects, with harmful effects at high concentrations and positive ones at low doses. Chronic high-dosage antioxidant supplementation decreases exercise-induced physiological adaptations and blocks redox-sensitive signaling pathways. The OS may result from increased ROS formation brought on by reductive stress. Given the tight relationship between inflammation and OS diseases, concerns regarding antioxidant therapy are raised.

Combination treatments using anti-inflammatory and antioxidant medications may be effective in treating male infertility due to OS. Nevertheless, the effect of IVF does not solely depend on OS. In vitro, the development of mature oocytes and embryos of high quality mainly depends on the quality of the oocytes per se as well as the microenvironment of follicular fluid. Thus, the delicate balance between OS and antioxidant activity is a determining factor in reproductive health.

## 12. Methodology

This review followed a structured approach to comprehensively analyze relevant literature on oxidative stress and antioxidants in male infertility. We conducted a database search using Web of Science, PubMed, and Scopus, focusing on studies published from January 2000 to October 2024.

Our search employed key terms such as “oxidative stress”, “antioxidants”, “inflammation”, and “human infertility”. We included only peer-reviewed research, selecting articles for their relevance to clinical or molecular insights on the role of antioxidants in human fertility. Studies using non-human models were excluded, except when their conclusions offered valuable insights applicable to human contexts. Additionally, we applied further exclusion criteria to articles lacking sufficient data to support their findings or displaying inadequate methodological transparency.

This approach enabled us to curate and synthesize findings from a representative body of work, emphasizing literature that directly addresses the interplay between oxidative stress, antioxidants, and human infertility.

## Figures and Tables

**Table 1:** Key Metabolic Pathways and Their Roles in Oxidative Stress and Male Infertility. The key processes that produce reactive oxygen species (ROS), their antioxidant defenses, and their effects on male reproductive health are described in this table. Varicocele and idiopathic male infertility are among the disorders that can result from abnormalities in these pathways, which can also lead to oxidative stress and impair sperm function.

Component/Metabolic Pathway	Function	Impact on Male Infertility
Respiratory Chain	Produces O_2_ and other reactive oxygen species (ROS)	controls cellular activity; excess ROS may impact cellular performance
Tricarboxylic Acid (TCA) cycle	Produces energy and generates ROS	ROS are essential for the creation of energy and can cause oxidative stress
Hydrogen Peroxide	metabolic byproduct that may result in the generation of OH•	Elevated levels can be harmful; require conversion to H_2_O
Catalase, GPx, Prx (Antioxidants)	Convert H_2_O_2_ into H_2_O	Prevent oxidative damage; excessive intake may result in an imbalance of GSH
Reduced Glutathione (GSH)	Necessary for GPx function	preserves redox state; excessive production may result in reductive stress
Methionine Cycle and Trans-sulfuration	Synthesizes cysteine, necessary for GSH formation	A surplus of GSH can upset redox equilibrium and affect antioxidant balance
Mitochondrial Complex I	Involved in NADH oxidation	Increased NADH and oxidative stress are caused by impaired function
Varicocele and Idiopathic Male Infertility	Conditions linked to oxidative stress and imbalance in redox status	Correlates with poor sperm motility and reproductive issues
PARP and TDP1	Involved in DNA repair processes	The integrity of sperm DNA depends on activity that is impacted by oxidative stress
Antioxidant Treatment	Used for diseases associated with oxidative stress	Improving sperm function has mixed results; supplementing should be done with caution

**Table 2:** Overview of catalase and superoxide dismutase (SOD) functions in ovarian physiology. Catalase and different SOD isoforms play important roles in controlling oxidative stress, follicular growth, and general reproductive health, as this table illustrates.

Component	Function	Expression	Impact on Follicular Health
Catalase	During meiosis, the metabolism of reactive oxygen species (ROS) shields the DNA from oxidative damage	Primarily located in peroxisomes; minimally expressed in oocytes. Higher activity in larger follicles of pigs, goats, and rats	Chromosome abnormalities may result from inhibition. Increased activity inhibits apoptosis during follicular maturation and is associated with FSH stimulation
Gonadotropins (FSH)	Control the steroidogenesis, differentiation, and maturation of follicles	Following FSH stimulation, catalase activity rises, particularly in larger follicles	Keeps the levels of steroids and ROS in balance and aids in follicle selection
Superoxide Dismutase (SOD)	Enzymatic protection against oxidative stress; controls the ratio of hydrogen peroxide (H_2_O_2_) to superoxide (O^2•−^)	Secondary follicles and theca cells contain SOD1 and SOD2, but large antral follicles have less activity	Vital for shielding oocytes from oxidative damage, activity rises during proestrus. Low rates of fertilization can result from hyperactivity
SOD1	Reduces oxidative damage caused by inflammation to luteal cells; linked to the release of progesterone	Expressed in the oocyte nucleus and highly active in the corpus luteum	Essential for preserving the proper ROS levels for ovulation and the growth of embryos
SOD2	Scavenges superoxide from the mitochondria and controls oxidative stress in oocytes	Cumulus cell-oocyte complexes (COCs) express this protein, which is found in mammalian oocytes	Protects oocytes from oxidative damage; essential for ovulation and fertilization

## Data Availability

Data is unavailable due to privacy or ethical restrictions.

## References

[1] Mandal M., Sarkar M., Khan A., Biswas M., Masi A., Rakwal R., Agrawal G.K., Srivastava A., Sarkar A. (2022). Reactive Oxygen Species (ROS) and Reactive Nitrogen Species (RNS) in plants– maintenance of structural individuality and functional blend. Adv. Redox Res..

[2] Zorov D.B., Juhaszova M., Sollott S.J. (2014). Mitochondrial Reactive Oxygen Species (ROS) and ROS-Induced ROS Release. Physiol. Rev..

[3] Juan C.A., Pérez De La Lastra J.M., Plou F.J., Pérez-Lebeña E. (2021). The Chemistry of Reactive Oxygen Species (ROS) Revisited: Outlining Their Role in Biological Macromolecules (DNA, Lipids and Proteins) and Induced Pathologies. Int. J. Mol. Sci..

[4] Agarwal A., Virk G., Ong C., du Plessis S.S. (2014). Effect of oxidative stress on male reproduction. World J. Men’s Health.

[5] Agarwal A., Mulgund A., Hamada A., Chyatte M.R. (2015). A unique view on male infertility around the globe. Reprod. Biol. Endocrinol. RBE.

[6] Naz R.K., Rajesh P.B. (2004). Role of tyrosine phosphorylation in sperm capacitation / acrosome reaction [No title found]. Reprod. Biol. Endocrinol..

[7] Aranda-Rivera A.K., Cruz-Gregorio A., Arancibia-Hernández Y.L., Hernández-Cruz E.Y., Pedraza-Chaverri J. (2022). RONS and Oxidative Stress: An Overview of Basic Concepts. Oxygen.

[8] Ndhlala A.R., Moyo M., Van Staden J. (2010). Natural Antioxidants: Fascinating or Mythical Biomolecules?. Molecules.

[9] Lobo V., Patil A., Phatak A., Chandra N. (2010). Free radicals, antioxidants and functional foods: Impact on human health. Pharmacogn. Rev..

[10] Walke G., Gaurkar S.S., Prasad R., Lohakare T., Wanjari M. (2023). The Impact of Oxidative Stress on Male Reproductive Function: Exploring the Role of Antioxidant Supplementation. Cureus.

[11] Sen S., Chakraborty R., Andreescu S., Hepel M. (2011). The Role of Antioxidants in Human Health. ACS Symposium Series [Internet].

[12] Kaltsas A. (2023). Oxidative Stress and Male Infertility: The Protective Role of Antioxidants. Medicina.

[13] Halliwell B. (2013). The antioxidant paradox: Less paradoxical now?. Br. J. Clin. Pharmacol..

[14] Warraich U.e.A., Hussain F., Kayani H.U.R. (2020). Aging—Oxidative stress, antioxidants and computational modeling. Heliyon.

[15] Vona R., Pallotta L., Cappelletti M., Severi C., Matarrese P. (2021). The Impact of Oxidative Stress in Human Pathology: Focus on Gastrointestinal Disorders. Antioxidants.

[16] Waddell J., Khatoon R., Kristian T. (2023). Cellular and Mitochondrial NAD Homeostasis in Health and Disease. Cells.

[17] Maldonado E., Morales-Pison S., Urbina F., Solari A. (2023). Aging Hallmarks and the Role of Oxidative Stress. Antioxidants.

[18] Ngo V., Duennwald M.L. (2022). Nrf2 and Oxidative Stress: A General Overview of Mechanisms and Implications in Human Disease. Antioxidants.

[19] Ponnampalam E.N., Kiani A., Santhiravel S., Holman B.W.B., Lauridsen C., Dunshea F.R. (2022). The Importance of Dietary Antioxidants on Oxidative Stress, Meat and Milk Production, and Their Preservative Aspects in Farm Animals: Antioxidant Action, Animal Health, and Product Quality—Invited Review. Animals.

[20] Jomova K., Makova M., Alomar S.Y., Alwasel S.H., Nepovimova E., Kuca K., Rhodes C.J., Valko M. (2022). Essential metals in health and disease. Chem. Biol. Interact..

[21] Pham-Huy L.A., He H., Pham-Huy C. (2008). Free radicals, antioxidants in disease and health. Int. J. Biomed. Sci. IJBS.

[22] Ménézo Y.J., Hazout A., Panteix G., Robert F., Rollet J., Cohen-Bacrie P., Chapuis F., Clément P., Benkhalifa M. (2007). Antioxidants to reduce sperm DNA fragmentation: An unexpected adverse effect. Reprod. BioMedicine Online.

[23] Henkel R., Sandhu I.S., Agarwal A. (2019). The excessive use of antioxidant therapy: A possible cause of male infertility?. Andrologia.

[24] Jomova K., Raptova R., Alomar S.Y., Alwasel S.H., Nepovimova E., Kuca K., Valko M. (2023). Reactive oxygen species, toxicity, oxidative stress, and antioxidants: Chronic diseases and aging. Arch. Toxicol..

[25] Singh A., Kukreti R., Saso L., Kukreti S. (2019). Oxidative Stress: A Key Modulator in Neurodegenerative Diseases. Molecules.

[26] Sun D., Gao W., Hu H., Zhou S. (2022). Why 90% of clinical drug development fails and how to improve it?. Acta Pharm. Sin. B.

[27] Stipanuk M.H., Ueki I. (2011). Dealing with methionine/homocysteine sulfur: Cysteine metabolism to taurine and inorganic sulfur. J. Inherit. Metab. Dis..

[28] Meulmeester F.L., Luo J., Martens L.G., Mills K., van Heemst D., Noordam R. (2022). Antioxidant Supplementation in Oxidative Stress-Related Diseases: What Have We Learned from Studies on Alpha-Tocopherol?. Antioxidants.

[29] Wang K., Gao Y., Wang C., Liang M., Liao Y., Hu K. (2022). Role of Oxidative Stress in Varicocele. Front. Genet..

[30] Rios J.S., Coward R.M., Hansen K.R., Barnhart K.T., Cedars M.I., Legro R.S., Diamond M.P., Krawetz S.A., Usadi R., Baker V.L. (2021). Sperm deoxyribonucleic acid fragmentation: Predictors, fertility outcomes, and assays among infertile males. FS Rep..

[31] Moritz L., Hammoud S.S. (2022). The Art of Packaging the Sperm Genome: Molecular and Structural Basis of the Histone-To-Protamine Exchange. Front. Endocrinol..

[32] Pommier Y., Nussenzweig A., Takeda S., Austin C. (2022). Human topoisomerases and their roles in genome stability and organization. Nat. Rev. Mol. Cell Biol..

[33] Tandara M., Bajić A., Tandara L., Bilić-Zulle L., Šunj M., Kozina V., Goluža T., Jukić M. (2014). Sperm DNA integrity testing: Big halo is a good predictor of embryo quality and pregnancy after conventional IVF. Andrology.

[34] Sadeghi N., Boissonneault G., Tavalaee M., Nasr-Esfahani M.H. (2023). Oxidative versus reductive stress: A delicate balance for sperm integrity. Syst. Biol. Reprod. Med..

[35] Andrés C.M.C., Pérez De La Lastra J.M., Juan C.A., Plou F.J., Pérez-Lebeña E. (2024). Antioxidant Metabolism Pathways in Vitamins, Polyphenols, and Selenium: Parallels and Divergences. Int. J. Mol. Sci..

[36] Kurutas E.B. (2015). The importance of antioxidants which play the role in cellular response against oxidative/nitrosative stress: Current state. Nutr. J..

[37] Agarwal A., Nallella K.P., Allamaneni S.S., Said T.M. (2004). Role of antioxidants in treatment of male infertility: An overview of the literature. Reprod. Biomed. Online.

[38] Ribeiro J.C., Braga P.C., Martins A.D., Silva B.M., Alves M.G., Oliveira P.F. (2021). Antioxidants Present in Reproductive Tract Fluids and Their Relevance for Fertility. Antioxidants.

[39] Zhou J., Fang C., Rong C., Luo T., Liu J., Zhang K. (2023). Reactive oxygen species-sensitive materials: A promising strategy for regulating inflammation and favoring tissue regeneration. Smart Mater. Med..

[40] De Lamirande E., Lamothe G., Villemure M. (2009). Control of superoxide and nitric oxide formation during human sperm capacitation. Free Radic. Biol. Med..

[41] Traber M.G., Stevens J.F. (2011). Vitamins C and E: Beneficial effects from a mechanistic perspective. Free Radic. Biol. Med..

[42] Yu B., Huang Z. (2015). Variations in Antioxidant Genes and Male Infertility. BioMed Res. Int..

[43] Crisol L., Matorras R., Aspichueta F., Expósito A., Hernández M.L., Ruiz-Larrea M.B., Mendoza R., Ruiz-Sanz J.I. (2012). Glutathione peroxidase activity in seminal plasma and its relationship to classical sperm parameters and in vitro fertilization-intracytoplasmic sperm injection outcome. Fertil. Steril..

[44] Wang S., He G., Chen M., Zuo T., Xu W., Liu X. (2017). The Role of Antioxidant Enzymes in the Ovaries. Oxidative Med. Cell. Longev..

[45] Ra K., Park S.C., Lee B.C. (2023). Female Reproductive Aging and Oxidative Stress: Mesenchymal Stem Cell Conditioned Medium as a Promising Antioxidant. Int. J. Mol. Sci..

[46] Lu J., Wang Z., Cao J., Chen Y., Dong Y. (2018). A novel and compact review on the role of oxidative stress in female reproduction. Reprod. Biol. Endocrinol..

[47] Kaltsas A., Zikopoulos A., Moustakli E., Zachariou A., Tsirka G., Tsiampali C., Palapela N., Sofikitis N., Dimitriadis F. (2023). The Silent Threat to Women’s Fertility: Uncovering the Devastating Effects of Oxidative Stress. Antioxidants.

[48] Wu Y., Huang J., Chen H., Tao H., He Y., Yang G., Zha Q., Lash G.E., Li P. (2023). Tumor-Derived Oxidative Stress Triggers Ovarian Follicle Loss in Breast Cancer. Am. J. Pathol..

[49] Ruder E.H., Hartman T.J., Blumberg J., Goldman M.B. (2008). Oxidative stress and antioxidants: Exposure and impact on female fertility. Hum. Reprod. Update.

[50] Agarwal A., Leisegang K., Majzoub A., Henkel R., Finelli R., Panner Selvam M.K., Tadros N., Parekh N., Ko E.Y., Cho C.-L. (2021). Utility of Antioxidants in the Treatment of Male Infertility: Clinical Guidelines Based on a Systematic Review and Analysis of Evidence. World J. Men’s Health.

[51] De Ligny W., Smits R.M., Mackenzie-Proctor R., Jordan V., Fleischer K., De Bruin J.P., Showell M.G. (2022). Antioxidants for male subfertility. Cochrane Database Syst. Rev..

[52] Jóźków P., Rossato M. (2017). The Impact of Intense Exercise on Semen Quality. Am. J. Men’s Health.

[53] Dutta S., Sengupta P., Roychoudhury S., Chakravarthi S., Wang C.W., Slama P. (2022). Antioxidant Paradox in Male Infertility: ‘A Blind Eye’ on Inflammation. Antioxidants.

[54] Sharifi-Rad M., Anil Kumar N.V., Zucca P., Varoni E.M., Dini L., Panzarini E., Rajkovic J., Fokou P.V.T., Azzini E., Peluso I. (2020). Lifestyle, Oxidative Stress, and Antioxidants: Back and Forth in the Pathophysiology of Chronic Diseases. Front. Physiol..

[55] Agarwal A., Aponte-Mellado A., Premkumar B.J., Shaman A., Gupta S. (2012). The effects of oxidative stress on female reproduction: A review. Reprod. Biol. Endocrinol..

[56] Showell M.G., Mackenzie-Proctor R., Jordan V., Hart R.J. (2020). Antioxidants for female subfertility. Cochrane Database Syst. Rev..

[57] Xu X., Liu A., Hu S., Ares I., Martínez-Larrañaga M.R., Wang X., Martínez M., Anadón A., Martínez M.-A. (2021). Synthetic phenolic antioxidants: Metabolism, hazards and mechanism of action. Food Chem..

[58] Yim E., Baquerizo Nole K.L., Tosti A. (2014). Contact Dermatitis Caused by Preservatives. Dermatitis.

[59] Sun Z., Gao R., Chen X., Liu X., Ding Y., Geng Y., Mu X., Liu T., Li F., Wang Y. (2021). Exposure to butylated hydroxytoluene compromises endometrial decidualization during early pregnancy. Environ. Sci. Pollut. Res..

[60] Yang L., Chen Y., Liu Y., Xing Y., Miao C., Zhao Y., Chang X., Zhang Q. (2021). The Role of Oxidative Stress and Natural Antioxidants in Ovarian Aging. Front. Pharmacol..

[61] Yan F., Zhao Q., Li Y., Zheng Z., Kong X., Shu C., Liu C., Shi Y. (2022). The role of oxidative stress in ovarian aging: A review. J. Ovarian Res..

[62] Regan S.L.P., Knight P.G., Yovich J.L., Leung Y., Arfuso F., Dharmarajan A. (2018). Granulosa Cell Apoptosis in the Ovarian Follicle—A Changing View. Front. Endocrinol..

[63] Wale P.L., Gardner D.K. (2016). The effects of chemical and physical factors on mammalian embryo culture and their importance for the practice of assisted human reproduction. Hum. Reprod. Update.

[64] Handy D.E., Loscalzo J. (2012). Redox Regulation of Mitochondrial Function. Antioxid. Redox Signal..

[65] Combelles C.M.H., Holick E.A., Paolella L.J., Walker D.C., Wu Q. (2010). Profiling of superoxide dismutase isoenzymes in compartments of the developing bovine antral follicles. Reproduction.

[66] Lisse T.S. (2020). Vitamin D Regulation of a SOD1-to-SOD2 Antioxidative Switch to Prevent Bone Cancer. Appl. Sci..

[67] Sasaki H., Hamatani T., Kamijo S., Iwai M., Kobanawa M., Ogawa S., Miyado K., Tanaka M. (2019). Impact of Oxidative Stress on Age-Associated Decline in Oocyte Developmental Competence. Front. Endocrinol..

[68] Almansa-Ordonez A., Bellido R., Vassena R., Barragan M., Zambelli F. (2020). Oxidative Stress in Reproduction: A Mitochondrial Perspective. Biology.

[69] Matzuk M.M., Dionne L., Guo Q., Kumar T.R., Lebovitz R.M. (1998). Ovarian Function in Superoxide Dismutase 1 and 2 Knockout Mice. Endocrinology.

[70] Pizarro B.M., Cordeiro A., Reginatto M.W., Campos S.P.C., Mancebo A.C.A., Areas P.C.F., Antunes R.A., Souza M.D.C.B., Oliveira K.J., Bloise F.F. (2020). Estradiol and Progesterone Levels are Related to Redox Status in the Follicular Fluid During in vitro Fertilization. J. Endocr. Soc..

[71] Kawaguchi S., Sakumoto R., Okuda K. (2013). Induction of the expressions of antioxidant enzymes by luteinizing hormone in the bovine corpus luteum. J. Reprod. Dev..

[72] Sadeghi H.M., Adeli I., Calina D., Docea A.O., Mousavi T., Daniali M., Nikfar S., Tsatsakis A., Abdollahi M. (2022). Polycystic Ovary Syndrome: A Comprehensive Review of Pathogenesis, Management, and Drug Repurposing. Int. J. Mol. Sci..

[73] Mannucci A., Argento F.R., Fini E., Coccia M.E., Taddei N., Becatti M., Fiorillo C. (2022). The Impact of Oxidative Stress in Male Infertility. Front. Mol. Biosci..

[74] Chen Y., Yang J., Zhang L. (2023). The Impact of Follicular Fluid Oxidative Stress Levels on the Outcomes of Assisted Reproductive Therapy. Antioxidants.

[75] Barati E., Nikzad H., Karimian M. (2020). Oxidative stress and male infertility: Current knowledge of pathophysiology and role of antioxidant therapy in disease management. Cell. Mol. Life Sci..

[76] Garg A., Kumaresan A., Ansari M. (2009). Effects of Hydrogen Peroxide (H_2_O_2_) on Fresh and Cryopreserved Buffalo Sperm Functions During Incubation at 37 °C In Vitro. Reprod. Domest. Anim..

